# Biopolymeric Anticorrosion Coatings from Cellulose Nanofibrils and Colloidal Lignin Particles

**DOI:** 10.1021/acsami.1c08274

**Published:** 2021-08-19

**Authors:** Arman Dastpak, Philip Ansell, Justin R. Searle, Mari Lundström, Benjamin P. Wilson

**Affiliations:** † Hydrometallurgy and Corrosion, Department of Chemical and Metallurgical Engineering (CMET), Aalto University, P.O. Box 16200, Aalto, Espoo FI-00076, Finland; ‡ Materials Research Centre, College of Engineering, Swansea University, Bay Campus, Crymlyn Burrow, Swansea SA1 8EN, Wales, U.K.; § SPECIFIC, College of Engineering, Swansea University, Bay Campus, Crymlyn Burrow, Swansea SA1 8EN, Wales, U.K.

**Keywords:** water-borne, electrophoretic deposition, galvanized steel, scanning vibrating electrode technique, electrochemical impedance spectroscopy

## Abstract

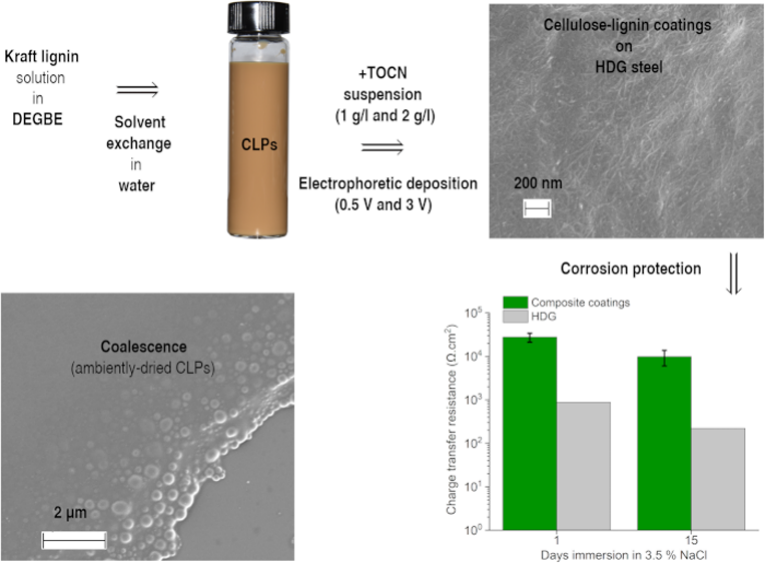

This study presents a process for preparation of cellulose–lignin barrier coatings for hot-dip galvanized (HDG) steel by aqueous electrophoretic deposition. Initially, a solution of softwood kraft lignin and diethylene glycol monobutyl ether was used to prepare an aqueous dispersion of colloidal lignin particles (CLPs) *via* solvent exchange. Analysis of the dispersion showed that it comprised submicron particles (*D* = 146 nm) with spherical morphologies and colloidal stability (ζ-potential = −40 mV). Following successful formation, the CLP dispersion was mixed with a suspension of TEMPO-oxidized cellulose nanofibers (TOCN, 1 and 2 g·L^–1^) at a fixed volumetric ratio (1:1, TOCN–CLPs), and biopolymers were deposited onto HDG steel surfaces at different potentials (0.5 and 3 V). The effects of these variables on coating formation, dry adhesion, and electrochemical properties (3.5% NaCl) were investigated. The scanning electron microscopy results showed that coalescence of CLPs occurs during the drying of composite coatings, resulting in formation of a barrier layer on HDG steel. The scanning vibrating electrode technique results demonstrated that the TOCN–CLP layers reduced the penetration of the electrolyte (3.5% NaCl) to the metal–coating interface for at least 48 h of immersion, with a more prolonged barrier performance for 3 V-deposited coatings. Additional electrochemical impedance spectroscopy studies showed that all four coatings provided increased levels of charge transfer resistance (*R*_ct_)—compared to bare HDG steel—although coatings deposited at a higher potential (3 V) and a higher TOCN concentration provided the maximum charge transfer resistance after 15 days of immersion (13.7 *cf.* 0.2 kΩ·cm^2^ for HDG steel). Overall, these results highlight the potential of TOCN–CLP biopolymeric composites as a basis for sustainable corrosion protection coatings.

## 1 Introduction

For more than a century, organic coatings and paints have been utilized effectively to protect metallic surfaces against corrosion.^1^ Nevertheless, this enduring popularity and corrosion protection functionality does not overshadow the predominant reliance of such materials on nonrenewable resources and their slow degradation/postservice accumulation as environmental pollutants.^2^ Consequently, there is an increasing need to develop new coating formulations based on sustainable resources, byproducts, or waste streams which have minimal impact on ecosystems during in-service and at the end-of-life stage.^3,4^

Lignocellulosic feedstocks are the most abundant biomass source obtained from plant matter that are primarily constituted of cellulose, hemicellulose, and lignin,^5^ altogether representing a potent renewable resource in the development of biobased economy and products.^6^ In the hierarchical structure of plants’ cell walls (*i.e.*, a natural composite), cellulose ultimately provides the structural scaffold,^7^ while lignin rigidifies the structure and provides a water-resistant barrier for the polysaccharide components.^8^ The inherent cofunctionality of cellulose and lignin in manmade composites has similarly demonstrated promising mechanical properties and barrier performance against water, water vapor, and oxygen permeation^9−13^—characteristics that also have the potential for application in corrosion protection. Nevertheless, these studies were mainly focused on preparation of free-standing films for applications such as ultraviolet (UV) shielding and packaging, while the anticorrosion performance of a cellulose–lignin coating has not, to the best of the authors’ knowledge, been investigated previously.

Cellulose nanofibrils (CNFs) are a nanoscale derivative of cellulose that are capable of forming dense film/coating networks (through intramolecular hydrogen bonds) with high mechanical properties and low oxygen permeability.^14,15^ To date, the functionality of CNFs as a barrier coating has primarily focused on paper/board applications,^16,17^ whereas its application for the protection of metallic surfaces is limited. The primary reason for this limitation could be due to the hydrophilic/hygroscopic nature of cellulose,^18^ although a few studies have exploited the water-swelling behavior of cellulose-containing coatings as a basis for release of corrosion inhibitors or healing agents.^19−22^

Lignin constitutes the main side stream of biomass-processing industries such as pulp paper and biorefineries.^23,24^ Currently, lignin’s potential as a feedstock for large-scale industrial processes cannot be fully realized due to its heterogeneous structure, high polydispersity, relatively low solubility in industrially utilized organic solvents, and its practical insolubility in water.^3,23,25^ One potential scalable and environmentally benign route to minimize these challenges is the preparation of water-dispersible colloidal lignin particles (CLPs) *via* nanoprecipitation/solvent exchange.^25−27^ The almost homogeneous colloidal particles prepared by this method are often spherical and demonstrate a negative surface charge, enabling them to maintain a reasonable stability as aqueous dispersions *via* electrical double layer repulsion,^28−30^ thereby facilitating the introduction of lignin into water-borne coating solutions.

The performance of lignin as the main component of anticorrosive coatings has so far received limited attention.^31^ In our previous studies,^32,33^ we have demonstrated the capability of unmodified lignin coatings to protect different steel grades; however, the coatings were solvent-borne, and their long-term protection performance (when immersed in corrosive electrolytes) was found to be limited. Following a different approach, this study—for the first time—aims to investigate the formation of CLPs from an industrial organic solvent (*i.e.*, diethylene glycol monobutyl ether, DEGBE) *via* solvent exchange in water for preparation of water-borne anticorrosion coatings.

Considering the negative charge of CLPs and TEMPO-oxidized CNFs (TOCN, a chemically pretreated derivative of CNFs with high water dispersibility^34^) in water, electrophoretic deposition (EPD) was utilized to investigate the effect of parameters (*e.g.*, applied potential and ratio of TOCN–CLP in the dispersion) on the deposition and the performance of fully biobased coatings on hot-dip galvanized (HDG) steel. The electrochemical properties and corrosion protection capabilities of these biopolymeric coatings were subsequently determined by both the scanning vibrating electrode technique (SVET) and electrochemical impedance spectroscopy (EIS). Moreover, the applicability of a common industrial organic solvent (DEGBE) for the formation of CLPs and its subsequent role during the film formation process is demonstrated. The findings of this study highlight the significant potential of water-borne biopolymeric coatings for the protection of HDG steel surfaces.

## 2 Experimental Section

### 2.1 Materials and Chemicals

The metal substrate investigated was a 0.54 mm thick commercial-grade HDG steel (FAX Z275, SSAB, Finland), which was cut into coupons (25 mm × 45 mm), cleaned by 10 min sonication in ethanol (94.2%, Altia, Finland) before being rinsed with deionized (DI) water. The biopolymers used for the coating material comprised previously characterized softwood kraft lignin (BioPiva 190, UPM, Finland)^33^ and a commercial TOCN gel (1 wt % solid content, 1.4 mmol COONa·g^–1^, Cellulose Lab, Canada). DEGBE (≥98%, Sigma-Aldrich, Germany) was selected for lignin dissolution, whereas sodium chloride (NaCl, ≥98%, Sigma-Aldrich, Germany) formed the basis for the (3.5%) electrolyte used in the SVET and EIS measurements. Initial monitoring of the pH gradient during the deposition process was done by application of a pH indicator solution (pH 4–10, Honeywell Fluka, USA). DI water with a resistance of ∼15 MΩ·cm was used throughout the experimental procedures.

### 2.2 Preparation of CLPs

Undried kraft lignin powder (0.75 g, KL) was added to DEGBE (5 mL) and left to stir at ambient temperature (∼21 °C) with a magnetic stirrer at 300 rpm (48 h) to form a 150 g·L^–1^ organic solution. This solution was subsequently centrifuged (5000 rpm/4612*g* force, 600 s, Thermo Fisher Scientific Heraeus Megafuge 16, Germany) to separate any insoluble residues. These residues were dried in a fume hood for 2 weeks to provide data for the solubility value calculation. Lignin solubility in DEGBE was determined to be 96.2% (±0.1%) based on an average of five gravimetric measurements of the insoluble constituents. CLPs’ formation involved the slow addition of 5 mL centrifuged lignin solution obtained at the center of a vortex in 50 mL of DI water, while the solution was magnetically stirred (300 rpm, 60 min). The resulting dispersion was centrifuged (2500 rpm/1153*g* force, 300 s) to separate any unstable colloidal particles present and was further used without the recovery/evaporation of DEGBE from the dispersion. The formation efficiency of the stable CLPs was obtained from triplicate gravimetric measurements *via* the following eq 1
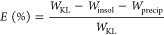
1where *W*_KL_ is the initial weight of undried KL, *W*_insol_ is the weight of insoluble KL in DEGBE, and *W*_precip_ is the weight of unstable CLPs that were precipitated during centrifugation.

### 2.3 Characterization of CLPs

Particle size measurements from two identical CLP dispersions were conducted using a laser diffraction analyzer equipped with a Hydro LV dispersion unit (Mastersizer 3000, Malvern, UK) using constant agitation rates (1800 rpm) in the dispersion unit and comparable laser obstruction levels (∼4%) for a total of 10 measurements. These measurements were conducted for both centrifuged and noncentrifuged CLP dispersions (Section 2.2).

To analyze the morphological features of CLPs, scanning electron microscopy (SEM, Zeiss Sigma VP, Germany) imaging was undertaken on dried lignin particles. Prior to imaging, multiple drops of the diluted dispersion (dilution factor = 200) were placed on a clean silicon wafer, which was then immediately placed in a preheated oven (180 °C, 30 min). Following oven treatment, the sample surface was sputter-coated (EM ACE600, Leica Microsystems, Germany) with iridium to deposit a ∼3 nm thick conductive layer and SEM surface micrographs were obtained using an in-lens detector with an acceleration voltage of 1 kV. Another set of surfaces was prepared following the same procedure, except that drying was carried out at ambient temperature (∼21 °C, 15 days).

Surface charge characteristics of CLPs in DI water were evaluated by ζ potential measurements (Zetasizer Nano ZS90, Malvern, UK). Measurements were conducted in triplicate with ∼2 mL of diluted dispersion (dilution factor = 50) after placing them in the measurement cell (ζ dip cell, ZEN1002). Calculation of the ζ-potential was performed automatically and was based on Smoluchowski theory.

### 2.4 Preparation of Coatings’ Medium and the Deposition Process

The EPD was conducted in a mixture of TOCN aqueous suspension and the CLP dispersion. TOCN suspensions were prepared by diluting the TOCN gel with DI water at two different ratios (1:10 and 1:5) to produce suspensions with solid concentrations of 1 and 2 g·L^–1^, respectively. These diluted cellulosic suspensions were then stirred at ambient temperature (∼21 °C) with a magnetic stirrer (300 rpm, 24 h) before being subjected to overhead shear-mixing for 15 min at 900 rpm (Eurostar 60) using a four-bladed propeller (R 1345, IKA, Germany). Once prepared, 50 mL of the TOCN suspension (either 1 or 2 g·L^–1^) was added to 50 mL of the centrifuged CLP dispersion (1:1 volumetric ratio), and the mixture (100 mL) was used directly for EPD. A maximum of four coatings were deposited from a single aqueous dispersion of biopolymers before it was replaced with a fresh dispersion.

The EPD process of the biopolymers was conducted using a conventional two-electrode setup with HDG steel as the anode and platinum (Pt) as the cathode. Furthermore, both electrodes had a rectangular geometry and the immersed area on each side of the HDG steel (6.25 cm^2^) was smaller than that of the platinum (8.28 cm^2^). A summary of the samples and parameters used for the EPD process is outlined in Table 1. After deposition, samples were left to dry horizontally in a fume hood (∼21 °C, 12 h) before they were placed in a preheated oven (105 °C, 1 h) to accelerate the solvent evaporation and to cure the coatings. A separate suspension—2 g·L^–1^ TOCN (without CLPs)—was used to prepare reference TOCN coatings at voltages of 0.5 and 3 V.

**Table 1:** Parameters Used for the EPD of TOCN–CLP Coatings on the HDG Substrate^a^

sample	*C*_TOCN_ (g·L^–1^)	*C*_CLPs_ (g·L^–1^)	*P* (V)	*T* (s)	*D* (mm)
0.1 T—0.5 V	1.0	11.2	0.5	150	10
0.2 T—0.5 V	2.0	11.2	0.5	150	10
0.1 T—3 V	1.0	11.2	3.0	150	10
0.2 T—3 V	2.0	11.2	3.0	150	10

a *C*_TOCN_ is the concentration of the TOCN suspension, *C*_CLPs_ is the concentration of the CLP dispersion, *P* is the deposition potential, *T* is the duration of deposition, and *D* is the distance between the anode and cathode.

### 2.5 Chemical Characterization of Coatings with ATR-FTIR

Attenuated total-reflectance (ATR) Fourier-transform infrared (FTIR) spectroscopy was used (Platinum-ATR, Bruker, USA) to study the chemical characteristics of the biopolymeric composite coatings. Surface measurements were conducted with a reflection diamond ATR-D cell to obtain 24 scans over a spectral range of 4000 to 750 cm^–1^. Additional measurements were performed on deposited TOCN coatings (lignin-free), a free-standing TOCN film, and dried CLP powder as a comparison. For the preparation of free-standing TOCN films and dried CLP powder, each suspension/dispersion was separately poured onto a watch glass and dried following a similar drying procedure used for the coatings (Section 2.4). Measurements of each sample were conducted in triplicate.

### 2.6 Investigation of Coating Morphology, Thickness, and Deposition Mass

The surface morphology of the coatings was investigated by SEM, and cross-sectional imaging was utilized to determine the coating thicknesses and to compare the cross-sectional morphology of the coatings. For thickness measurements, the coated substrates were vertically mounted in an epoxy resin, which after hardening (24 h) were cut in the center of the coated area. These samples were then sequentially polished, first with SiC paper (#320 to #1200 grit) and then with polycrystalline diamond suspensions (3 and 1 μm, Struers) before a final sonication in ethanol (94.2%, Altia) to clean the surface. Additionally, a separate set of coatings was prepared (according to Table 1) on HDG steel with a sealed bottom edge (1 mm × 25 mm area) using an electrical insulation tape (Temflex 1500, 3M, USA). This was to ensure that the morphological features of the coating cross sections were not damaged during the SEM preparation procedure. Sputter-coating with iridium for all cross-sectional samples was undertaken prior to SEM imaging (Section 3.2). Measurement of the coating thickness from individual SEM micrographs was conducted using ImageJ software [National Institutes of Health (NIH), Bethesda, USA].

Determination of the deposition mass was based on gravimetric measurements of the coated substrates after drying (*cf.* mass of blank substrate). This was achieved using a high-precision balance (0.01 mg, XSE202, Mettler Toledo, USA) with an average of 10 coatings for each sample condition (Table 1).

### 2.7 Characterization of Coatings’ Adhesion

The adhesion capability of composite coatings was investigated by pull-off adhesion tests. Measurements were conducted using a cross-hatch cutter (X2001, Paint Test Equipment, UK) with a multiblade (six parallel blades with 1 mm spacing) to apply scratches on the surface of coatings. An adhesive tape (XA001, Paint Test Equipment, UK) was then applied before being removed after 5 min of contact with the coating. This procedure was based on the ISO 2409 standard,^35^ which comprises a 0–5 grading system for coating adhesion, where 0 represents the best adhesion performance with no coating detachment proximal to the scored surface and 5 represents an adhesion failure where more than 65% of the coating area has detached from the substrate.

### 2.8 Electrochemical Characterization of Coated HDG Steel

The presence of local discontinuities in coatings (*i.e.*, pores/defects) was determined by SVET using an in-house assembled equipment based on a three-dimensional orthogonal motor-driven linear bearing array (Time and Precision Ltd., UK), with custom software-controlled movement and data logging (Swansea Innovations, Swansea University, Wales UK).^36,37^ The SVET probe consisted of a platinum wire microtip (125 μm in diameter) enclosed within a glass-capillary tube, which was vibrated at a frequency of 140 Hz, a 25 μm amplitude, and scanned at a height of 100 μm above the surface of immersed samples (3.5% NaCl). Each scan consisted of a 7 mm × 7 mm rastered area (6 mm × 6 mm for blank HDG steel) with over 8100 points and data from individual samples, obtained at 60 min intervals for a total duration of 72 h.

Coating corrosion protection capabilities were further investigated by EIS measurements. The EIS measurements were carried out using a potentiostat (IviumStat XRe, Ivium Technologies, The Netherlands) following exposure of a fixed area (0.785 cm^2^) on the blank HDG steel/coated surface to 300 mL of the electrolyte (3.5% NaCl). All the measurements were conducted inside a Faraday cage at ambient temperature (∼21 °C) using a three-electrode setup. The working electrode comprised the sample under investigation, and platinum was used as the counter electrode. The reference electrode (RE) was formed of an Ag/AgCl (saturated KCl) and a platinum (Pt) wire that were connected in parallel with a capacitor (100 nF) to form a dual RE. This was used to limit any high-frequency measurement errors that occur due to the resistance of the RE (in this case Ag/AgCl).^38^ EIS measurements were performed at open circuit potentials over a frequency range of 10 kHz to 0.01 Hz (logarithmically spaced with 12 steps/decade) with a signal amplitude of 10 mV_rms_ following 1 h exposure of surfaces to the electrolyte. Data from each sample were obtained at 1 day intervals over 15 days exposure to the electrolyte, and the electrolyte was substituted with a fresh solution every 7 days. The analysis of EIS data was achieved by using ZView software (Scribner Associates Inc., USA).

## 3 Results and Discussion

### 3.1 Formation and Characteristics of CLPs

**Figure 1:**
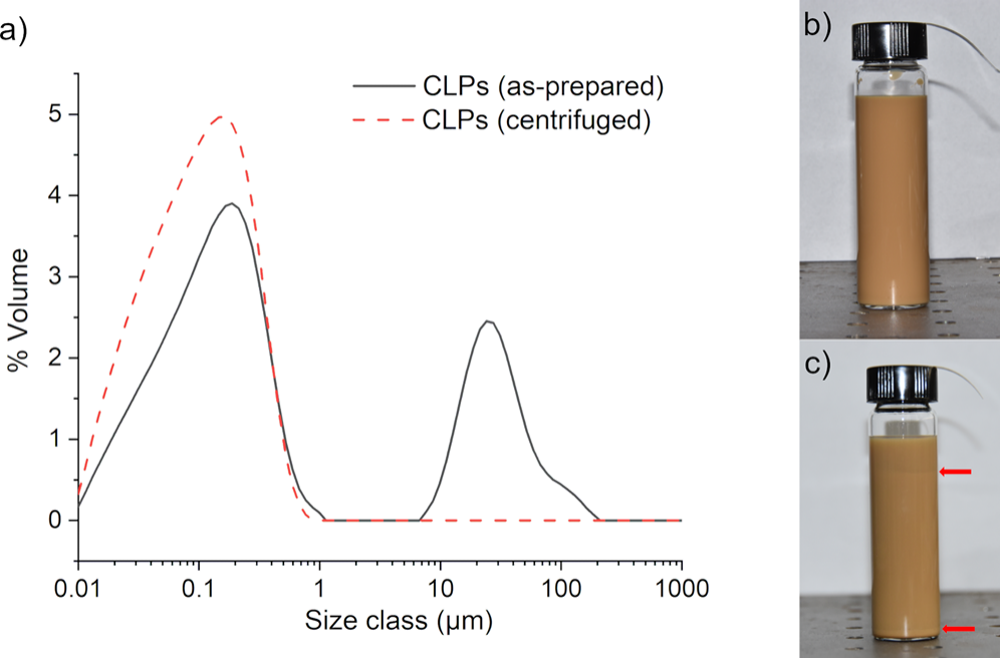
(a) Particle size distribution of CLPs before and after centrifugation. (b) Appearance of centrifuged CLPs immediately following preparation and (c) after 30 days storage. The red arrows in (c) indicate the boundary regions where particles appeared to be separated.

Figure 1a displays the size distribution of CLPs obtained by light scattering from prepared and centrifuged CLP dispersions. The graph obtained from the as-prepared CLPs demonstrates a bimodal distribution of fine and large particles with a volume-weighted mean of *D*(3,4) = 11.1 μm. Upon centrifugation with ∼1150*g* force for 300 s, the larger lignin particles settled down and the remaining dispersion demonstrates a relatively wide distribution of submicron particles with *D*(3,4) = 146 nm (Figure 1a). The presence of large particles in the initial CLP dispersion was most probably due to the high concentration of lignin in the stock solution (150 g·L^–1^), which resulted in the particle aggregation during the formation of CLPs.^29^ Nevertheless, the conversion efficiency (technical lignin to colloidally stable submicron particles) was ∼82%.

Visual inspection of the postcentrifugation dispersions showed high colloidal stability (Figure 1b), although with prolonged aging, the colloidal stability gradually decreased such that after 30 days, distinct regions of separation could be observed (Figure 1c). Furthermore, the ζ potential of CLPs was also found to reduce from an initial value of −40.5 ± 2.4 to −31.8 ± 3.5 mV after 30 days of aging, further indicating that stability decreases during storage. This loss of stability in dispersion of CLPs could have resulted from further interaction of particles, either through their fusion (*i.e.*, irreversible) and/or their aggregation (typically reversible).^39^ The presence of the residual solvent affects the dispersion stability by enabling the particle growth, for example, through Ostwald ripening, especially in colloidal systems with a wide size distribution of particles.^40,41^ This might explain the appearance of the separation layers in the aged dispersion (Figure 1c), although aggregation of particles or their coalescence upon collision might have also contributed to the observed changes in the dispersion’s stability.^39^ The outlined ζ potential values, nevertheless, are comparable to previously reported values for CLPs produced from softwood KL^28,42^—thereby demonstrating that DEGBE can be used as a solvent for formation of spherical CLPs. However, as the postpreparation recovery of the organic solvent is the most energy-demanding step of the whole CLP production process,^29^ use of DEGBE—which is a high-boiling-point solvent (∼230 °C)—may not offer sufficient advantages as an alternative solvent, especially for thermal-based recovery methods. In the case of this study, the presence of DEGBE within the CLP dispersions was found to be necessary as it plays a crucial role during the film-formation process.

**Figure 2:**
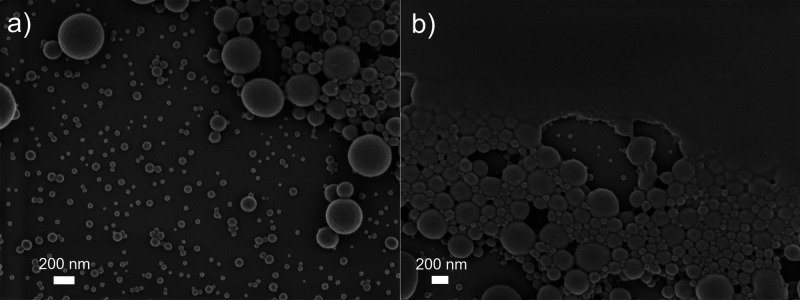
SEM micrographs of dried CLPs on a silicon substrate, (a) demonstrating the spherical morphology of CLPs and (b) presence of a coalesced region on the substrate.

Figure 2a,b displays SEM micrographs obtained from the colloidal particles subjected to relatively fast drying (180 °C, 30 min) after application onto the silicon substrate surface. Figure 2a clearly shows the spherical morphology of the dried colloidal particles and their wide size distribution, confirming the results from the laser diffraction measurements. Moreover, it can be observed that a region of coalesced particles also exists on the dried surfaces (Figure 2b), where CLPs have formed a more continuous film rather than a collection of closely packed individual particles. This probably results from the presence of DEGBE which has a coalescing effect on lignin particles,^43^ similar to some other slow-evaporating organic solvents that are utilized as coalescing additives in waterborne latex paints.^44^ Furthermore, the SEM micrographs of ambiently dried surfaces also showed a continuous film of coalesced CLPs (Figure S1, Supporting Information), indicating that the coalescence of CLPs is due to the presence of DEGBE rather than the heat-treatment process. Consequently, DEGBE provides a dual functionality, first, as the solvating medium for KL prior to CLP formation^33^ and second, as a coalescing agent during the film formation process.

### 3.2 EPD and the Formation of TOCN–CLP Coatings

**Figure 3:**
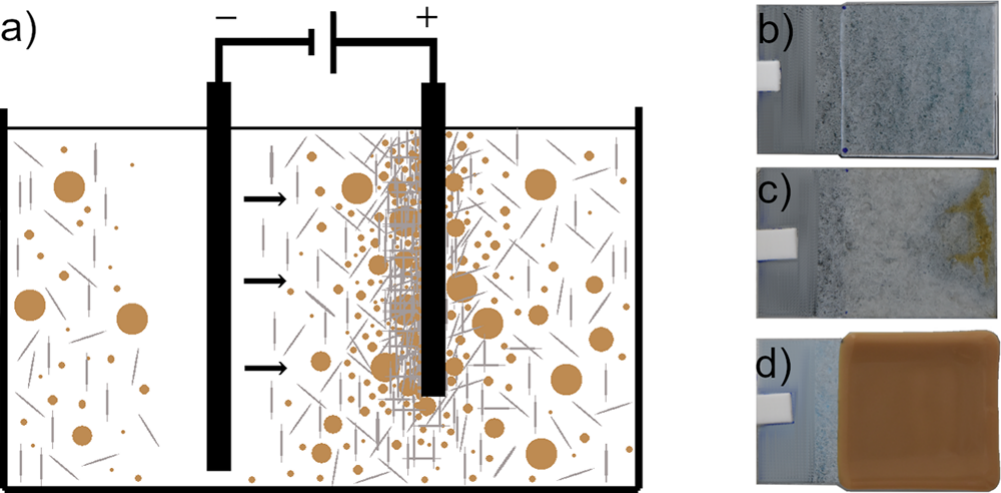
(a) Schematic of the electrophoretic codeposition of negatively charged CLPs (brown spheres) and TOCN (gray lines) from aqueous dispersions onto a positively charged HDG steel surface, (b) appearance of the coating deposited from TOCN suspensions, (c) substrate deposited from CLP dispersion, and (d) coating obtained from codispersion of TOCN and CLPs. Deposition parameters (3 V, 150 s) and concentration of biopolymers in the dispersions were fixed (b–d). The dimension of each substrate is 4 cm × 2.5 cm, and the deposition scheme (a) is not scaled.

Figure 3a outlines a general schematic for the simultaneous EPD of TOCN and CLPs. Due to the negative net charge of both biopolymers dispersed/suspended in water—with measured ζ-potentials = −40.5 ± 2.4 mV (CLPs) and −45.4 ± 1.2 mV (TOCN)—application of an external DC electric field initiates the migration of negatively charged biopolymers toward the positively charged HDG substrate. This is followed by the charge neutralization and deposition of materials on the anode surface.^45^ Initial investigations of the two biopolymers separately demonstrated that deposition from the TOCN suspension (without CLPs) was achievable (Figure 3b), whereas the same deposition parameters from the CLP dispersion (without TOCN) did not yield a discernible deposit (Figure 3c). In contrast, the presence of the two biopolymers within the dispersion resulted in the deposition of layers with the highest apparent thickness (Figure 3d). Furthermore, the average current density values during the codeposition of biopolymers were higher than those obtained from the deposition of individual components (Figure S2, Supporting Information).

To understand the mechanism of the formation of the biopolymeric deposit, the effect of experimental parameters on the charge neutralization of TOCN must be considered. During the EPD process, the electrolysis of water could take place, which would result in a localized drop of pH proximal to the anode (due to oxygen evolution) and increased pH at the cathode (hydrogen evolution), according to the following reactions 2 and 3(46)
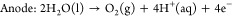
2
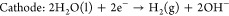
3

However, considering the sacrificial nature of zinc present on the HDG surface, a competing anodic reaction, that is, dissolution of zinc, can also exist during the deposition process according to reaction 4(47)
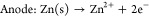
4

One possible mechanism for deposition is that the oxidation reaction at the anode leads to the charge neutralization of the sodium carboxylate groups (COO^–^Na^+^) present within TOCN. The products of the oxidation reaction at the anode cause a drop in local pH (reaction 2), resulting in the dissociation of sodium counter ions and a subsequent formation of carboxylic acid (COOH) groups on the surface of nanofibrils, which in turn induces a sol–gel transition and results in formation of the surface deposit.^46^ Alternatively, the sol–gel transition could be induced by metal cations (in this case Zn^2+^) that are released by the anodic dissolution of zinc during the deposition that cross-link with TOCN carboxylate groups.^48,49^ This mechanism of hydrogel formation has been previously reported in the literature to occur with divalent (Zn^2+^, Cu^2+^, and Ca^2+^) and trivalent cations (Al^3+^ and Fe^3+^).^50,51^

In contrast, with a suspension that contains only CLPs, there is no similar mechanism that allows for the formation of a readily stable deposit, as exhibited by the lack of CLPs apparent on the surface under the same EPD conditions (Figure 3c). Nonetheless, when the deposition is conducted from a dispersion containing both TOCN and CLPs, the formation of the TOCN hydrogel on the surface assists the immobilization of CLPs within the cellulosic network, resulting in the formation of TOCN–CLP coatings.^46^ These results suggest that the main anodic reaction occurring during the deposition is the dissolution of zinc from the HDG anode (rather than evolution of oxygen) and that the hydrogel formation is induced by the metal cations and not the formation of carboxylic acid moieties. This is supported by the observation that only a minor pH change occurs at the HDG anode versus relatively high cathodic hydrogen evolution during the deposition (Figure S3, Supporting Information) and that only relatively small voltages are used in the deposition process; therefore, the driving force for reaction 2 to occur is low.

**Figure 4:**
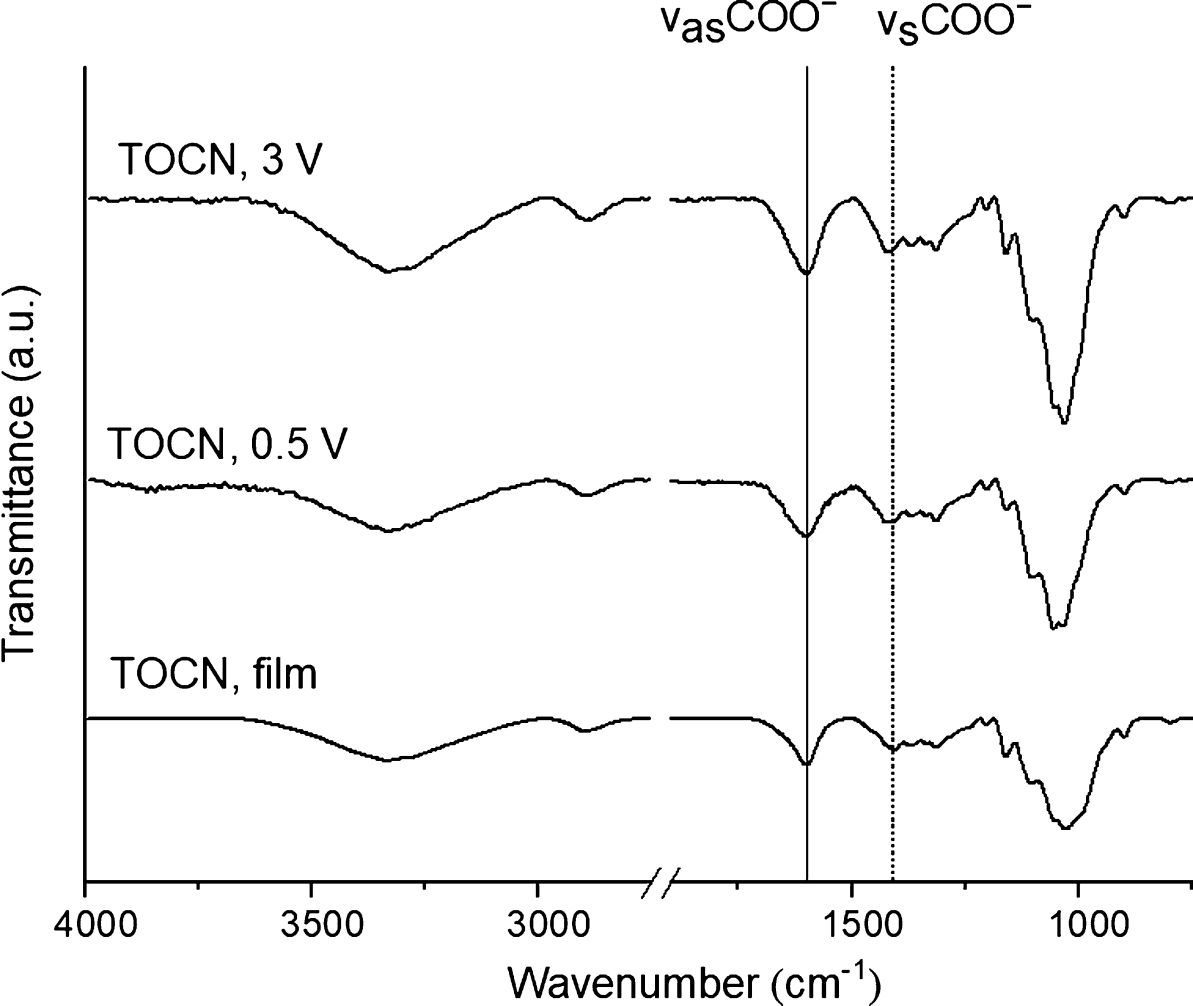
ATR-FTIR transmittance spectra for the TOCN free-standing film and coated samples on HDG surfaces. The spectrum of the free-standing film is normalized to the peak at 1033 cm^–1^.

ATR-FTIR characterization of coatings further clarifies the mechanism of deposit formation. Figure 4 displays the spectral characteristic of TOCN coatings (deposited at 0.5 and 3 V) and that of a free-standing TOCN film as a reference. As can be observed, the positions of typical bands of TOCN for the stretching vibration of −OH (∼3330 cm^–1^), stretching vibration of C–H (∼2900 cm^–1^), and −OCO– asymmetric vibration band of carboxylate anions (∼1600 cm^–1^) are identical for all samples. Conversely, the band for the −OCO– symmetric vibration of the carboxylate anions within the coatings (∼1419 cm^–1^) shifts to higher wavenumbers when compared to that present in the free-standing film (∼1409 cm^–1^), which suggests that the carboxylate anions in the coatings have formed a new complex with Zn cations.^50^ Furthermore, if the assumption is that deposit formation is based on the creation of carboxylic acid moieties, a new band (∼1735 cm^–1^) should be present within the spectra of TOCN coatings; however, this is clearly not the case.^46,52^

**Figure 5:**
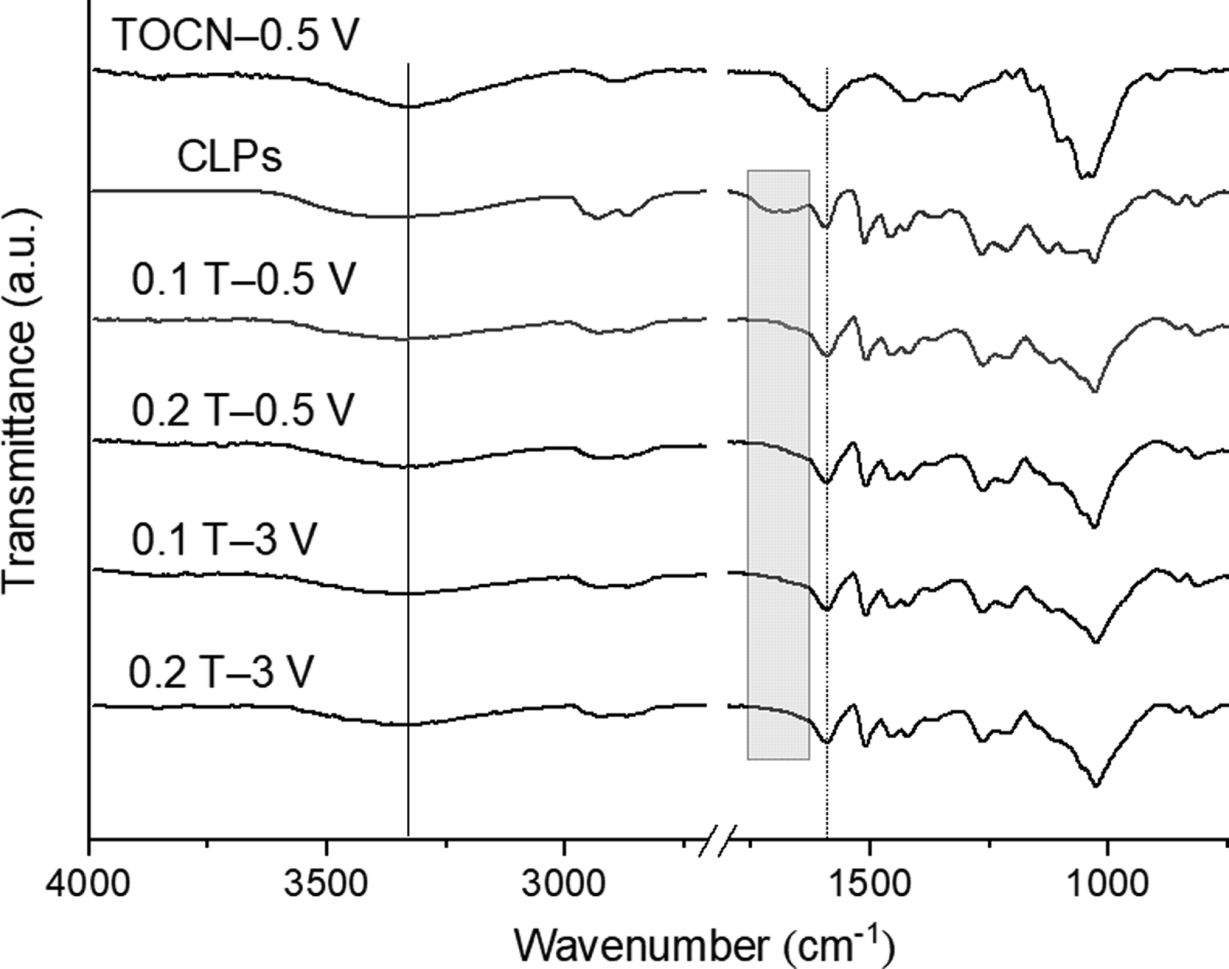
ATR-FTIR transmittance spectra for the reference TOCN coating, powdered CLPs, and composite coatings.

Figure 5 shows the transmittance FTIR spectra of composite coatings (TOCN–CLPs) alongside CLP powder and TOCN reference coating (deposited at 0.5 V). As can be observed, the composite coating spectral characteristics resemble those for both CLPs and TOCN references with no significant band position changes observable—except for the disappearance of the wide band in CLPs at ∼1700 cm^–1^ that relates to unconjugated −OCO– group stretching.^53^ When compared to the spectra recorded for the composites and the TOCN alone (outlined by the gray box in Figure 5), there is a slight shift in the band position of TOCNs’ −OCO– asymmetric vibration (∼1600 cm^–1^) to lower wavenumber (∼1591 cm^–1^) in the coatings. Similarly, the band corresponding to the stretching vibration of −OH in CLPs (∼3370 cm^–1^) appears at lower wavenumbers (∼3330 cm^–1^) for the composite coatings. Therefore, it could be speculated that the main interaction between TOCN and CLPs occurs through hydrogen bonding,^53^ which is one of the primary interaction routes between cellulose and lignin that is also found in the native cell wall of biomass.^54,55^ Nevertheless, due to the overlapping spectral features from the two biopolymers—specifically the −OCO– asymmetric vibration of TOCN with the aromatic skeletal vibration of lignin at ∼1600 cm^–1^^56^—utilization of more targeted characterization techniques would be required to comprehensively elucidate the nature of bonding between the two main components within the composite coatings.

### 3.3 Deposition Yield and Morphology of Coatings

**Figure 6:**
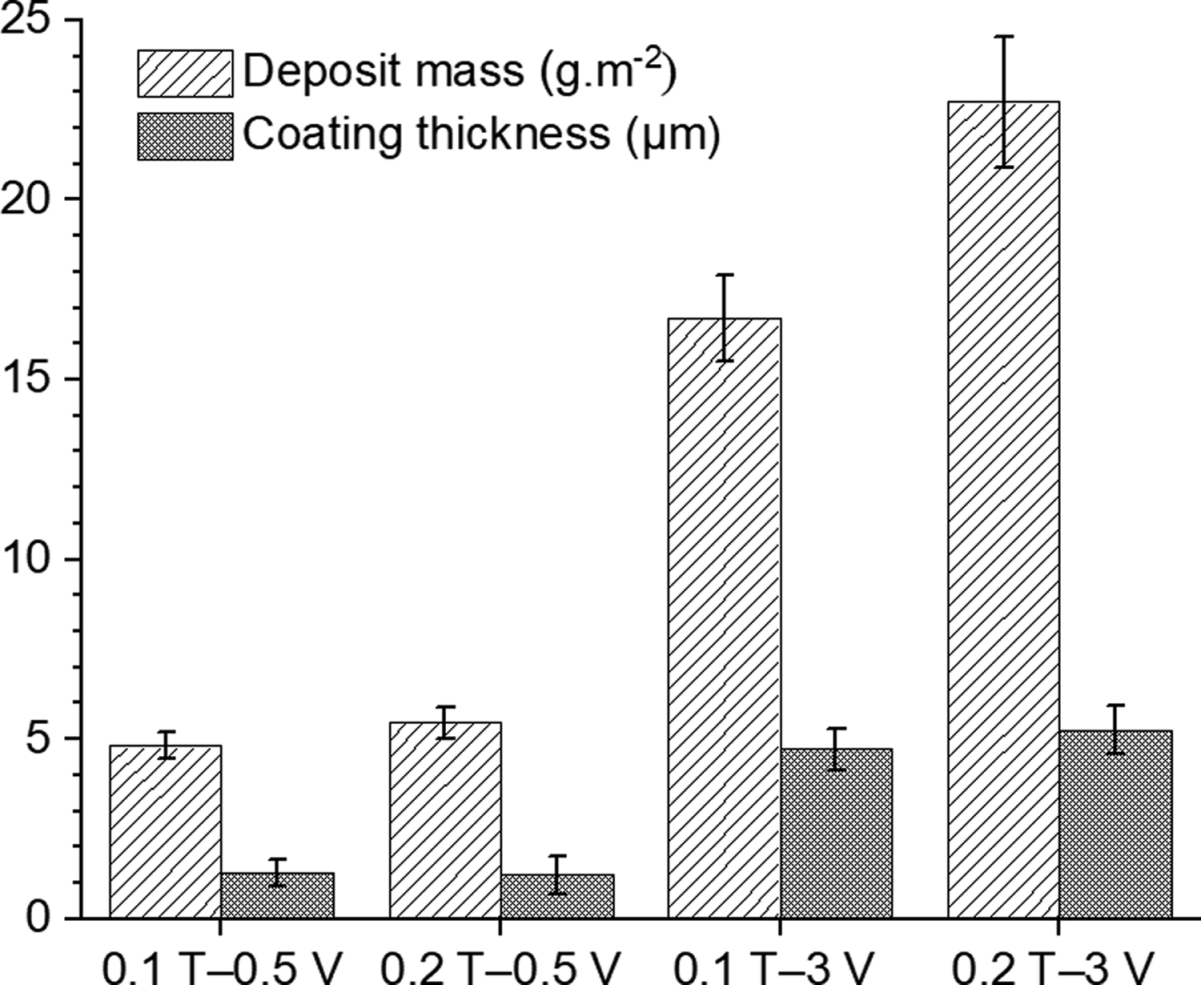
Deposited mass and coating thicknesses of composite coatings obtained at different TOCN concentrations and deposition potentials.

The mass of deposits and the measured thickness of coatings are illustrated in Figure 6. As expected,^46^ a direct correlation exists between the deposited mass and the initial concentration of TOCN in the dispersions, especially for coatings deposited at the higher potential of 3 V. Therefore, it can be concluded that an increase in both the initial concentration of TOCN and the deposition voltage results in a higher deposition yield, which produces thicker coatings. For example, while the deposition at 0.5 V for both TOCN concentrations (1 and 2 g·L^–1^) results in comparable coating thicknesses of 1.3 ± 0.4 μm (0.1 T—0.5 V) and 1.2 ± 0.5 μm (0.2 T—0.5 V), a change of the deposition potential to 3 V significantly increases the resultant coating thicknesses to 4.7 ± 0.6 μm (0.1 T—3 V) and 5.3 ± 0.6 μm (0.2 T—3 V). It is worth noting that in EPD, the deposition voltage and the initial concentration of materials are the primary parameters that can be used to control the mass of the deposit and therefore, the resultant coating thickness.^45^

**Figure 7:**
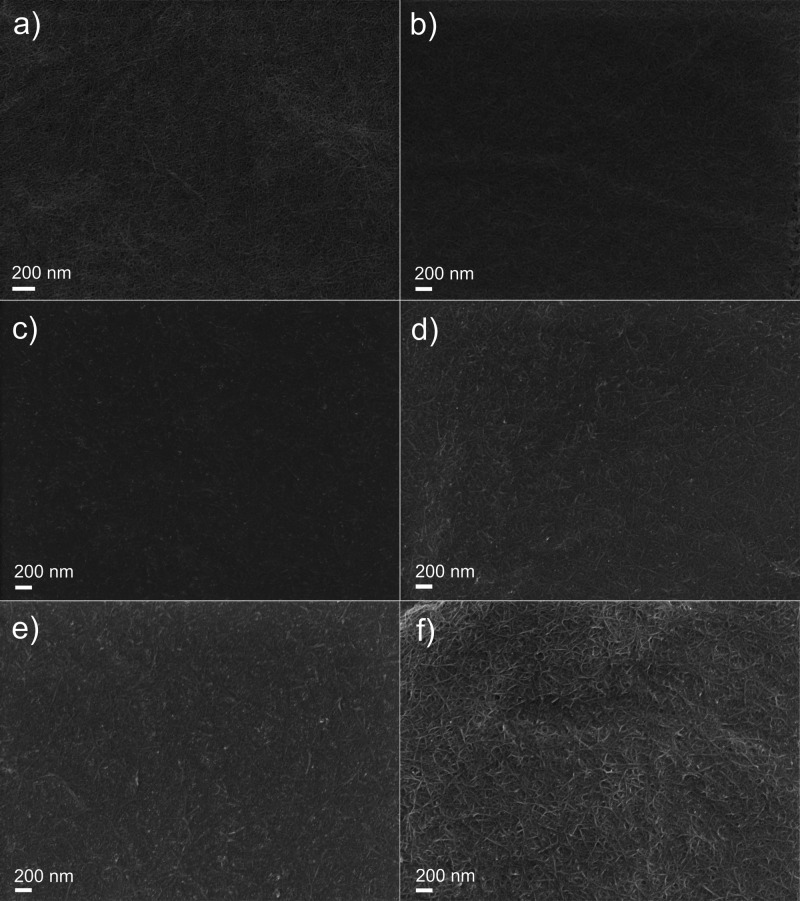
SEM micrographs obtained from the coating surfaces: (a) 1 g·L^–1^ TOCN—3 V, (b) 2 g·L^–1^ TOCN—3 V, (c) 0.1 T—0.5 V, (d) 0.2 T—0.5 V, (e) 0.1 T—3 V, and (f) 0.2 T—3 V.

Figure 7 displays SEM micrographs obtained from the surface of composite coatings (listed in Table 1) and TOCN reference coatings deposited from 1 to 2 g·L^–1^ TOCN suspensions at 3 V. For TOCN coatings (Figure 7a,b), the surfaces appear to comprise a random fibrillar network of cellulose that is aligned parallel to the surface of HDG steel and features small voids that are due to the porous nature of CLP-free cellulosic coatings. On the other hand, the TOCN–CLP composite coatings (Figure 7c–f) show relatively compact networks of nanofibrils that are covered with coalesced CLPs. Additionally, the concentration of TOCN on the surface appears to be greater for coatings deposited from the higher concentration of TOCN dispersions (2 g·L^–1^, Figure 7d,f), and this effect is more noticeable for 3 V-deposited coatings (Figure 7f). Furthermore, a small number of cracks was observed in the surface micrographs of all coatings, although these were more noticeable for coatings deposited at higher voltages, that is, 3 V (Figure S4, Supporting Information).

**Figure 8:**
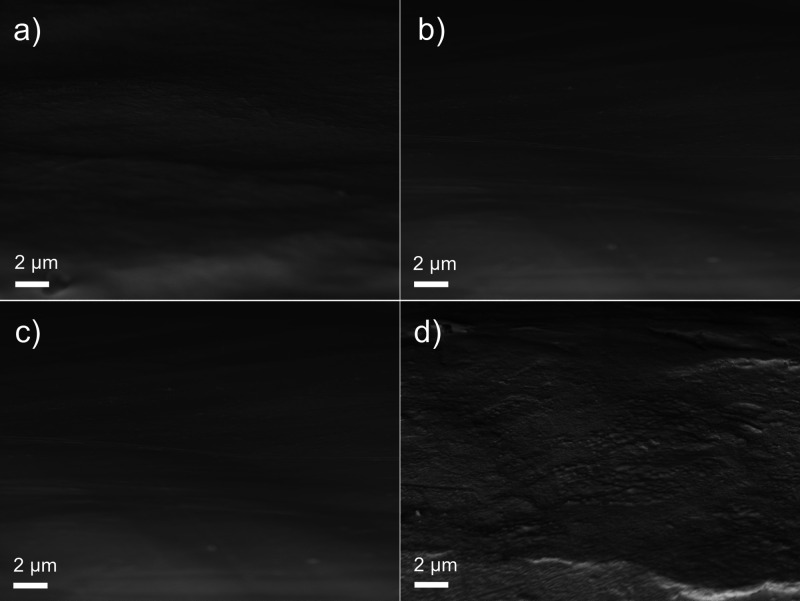
Cross-sectional SEM micrographs obtained from coatings: (a) 0.1 T—0.5 V, (b) 0.2 T—0.5 V, (c) 0.1 T—3 V, and (d) 0.2 T—3 V.

Figure 8 shows the SEM micrographs obtained from the cross sections of composite coatings. Following a similar trend as the surface morphology (Figure 7), the coalescence of CLPs occurs consistently throughout coatings and is not a surface-specific phenomenon. Furthermore, an integrated network of TOCN–CLPs appears to form (Figure 8a–d) in all the cross sections, suggesting that the local CLP agglomeration does not occur during film formation in the composite coatings.

### 3.4 Adhesion of Composite Coatings to HDG Steel

**Figure 9:**
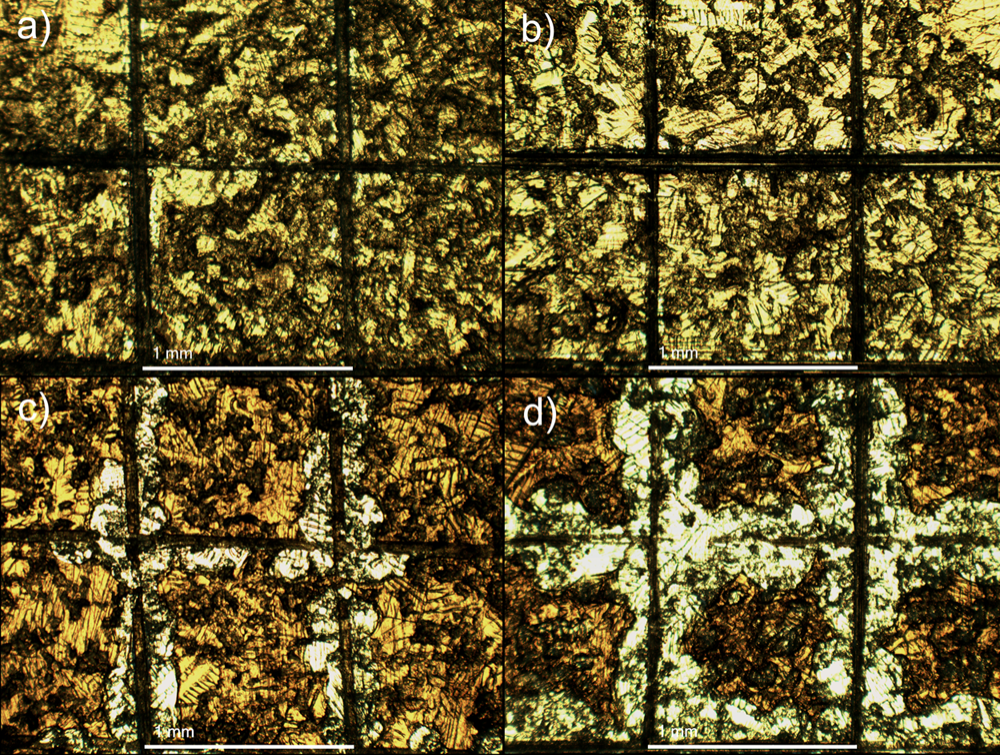
Optical microscopy images obtained from the surface of coatings after cross-cut measurements: (a) 0.1 T—0.5 V, (b) 0.2 T—0.5 V, (c) 0.1 T—3 V, and (d) 0.2 T—3 V. (Scale bar: 1 mm).

The adherence of TOCN–CLP coatings to the HDG surface was investigated by cross-cut adhesion measurements. Table 2 lists the quantified estimation for the coatings’ adhesion, which according to ISO 2409 is based on the degree of coating detachment from the scored area.^35^ As demonstrated in Figure 9, the samples deposited at 0.5 V (Figure 9a,b) have smooth cut edges without any coating detachment evident; in contrast, the coatings deposited at 3 V (Figure 9c,d) have lost a higher percentage of the coating from the incision area. Therefore, according to ISO 2409, the adhesion of composite coatings deposited at 3 V is inferior to that of 0.5 V-deposited samples. Nevertheless, it appears for 3 V coatings that the main portion of coating detachment occurs during the cutting procedure, rather than after the application and removal of the adhesive tape (Figure S5, Supporting Information), which suggests increased residual stress in the coatings due to the changes in the TOCN concentration and deposition voltage, as the residual stress in a coating is known to adversely affect the adhesion properties.^57^

**Table 2:** Quantified Values for Adhesion Performance of Composite Coatings

coating	0.1 T—0.5 V	0.2 T—0.5 V	0.1 T—3 V	0.2 T—3 V
adhesion	0	0	3	4

According to the ISO 2409 standard, the adhesion value of 0 represents the best performance without any detachment of the coating in the test area and 5 represents the adhesion failure (>65% detachment of coatings).^35^

To understand the observed difference in the cross-cut measurements of coatings, different stages of drying must be considered. Generally, the drying of a polymeric coating is associated with the development of an in-plane tensile stress. These stresses result from the adhesion of a coating to the substrate, which constrains the three-dimensional shrinkage across the coating’s network.^58^ When the TOCN and CLP components of the studied coating are considered, it is reasonable to assume that the initial drying stages involve a relatively fast evaporation of water that results in the solidification and formation of a TOCN lattice with an associated level of shrinkage.^59^ In contrast, the coalescence of CLPs and the related slow evaporation of the organic solvent only occur during the latter stages of drying. In scenarios where the formation of a solid network occurs (in this case TOCN) before the evaporation of the organic solvent, an increase in the coating thickness enhances the level of the accumulated stress in the final dried coating as the diffusion of solvent molecules throughout the coating is constricted by a solidified network.^60^ However, a higher extent of the residual stress in 3 V coatings could also be attributed to the increased content of CLPs (Figure 6) as a function of deposition potential (due to faster kinetics of deposition^44^) and TOCN concentration (higher immobilization of CLPs in the TOCN network^46^). As such, the increased content of CLPs (with a wide size distribution) could increase the capillary pressures during the solidification of coating and might further resist the relaxation of the stress due to the coalescence effect.^61^ Nevertheless, this hypothesis could not be confirmed as either the development of stress during the drying procedure or the glass transition temperature of CLPs were beyond the scope of this study.

### 3.5 Electrochemical Characterization of Coated HDG Steel

The SVET provides a map of the current density distribution above an electrochemically active—usually corroding— surface when immersed in an electrolyte. From such maps, the local anodic and cathodic sites can be identified and their evolution over time can be characterized. The presence of organic coatings on a metal surface generally impede the measurement of ionic current produced beneath the coating by the SVET, although areas of electrochemical activity can be detected through coating defects or pores, should they be sufficiently large (μm-scale) to induce current paths at the level of the probe scan height.^62,63^ Consequently, in this case, the SVET was used as a method to study the overall homogeneity of the deposited coatings and spatial distribution of corrosion activity that occurs during immersion in a corrosive electrolyte due to the presence of local surface discontinuities (*i.e.*, pores/defects).

**Figure 10:**
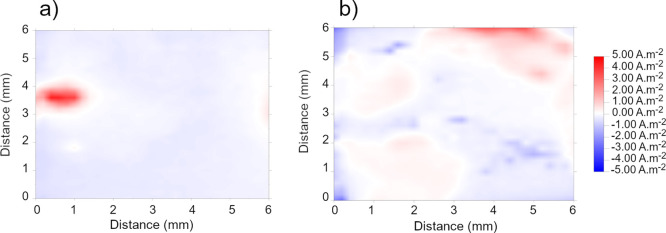
SVET’s map of ionic current over bare HDG steel after immersion in 3.5% NaCl for (a) 2 and (b) 72 h.

Figure 10 illustrates the ionic current density map obtained over the surface of bare HDG steel (72 h in 3.5% NaCl). During the initial stages of immersion (2 h, Figure 10a), the electrochemical activity manifests as a relatively focused anodic site (dissolution of zinc) that is surrounded by a cathodic region. Over a prolonged immersion time of 72 h, the anodic activity spreads across the whole surface due to the formation of additional anodic sites and the local current density decreases due to the formation of an oxide layer (Figure 10b).

**Figure 11:**
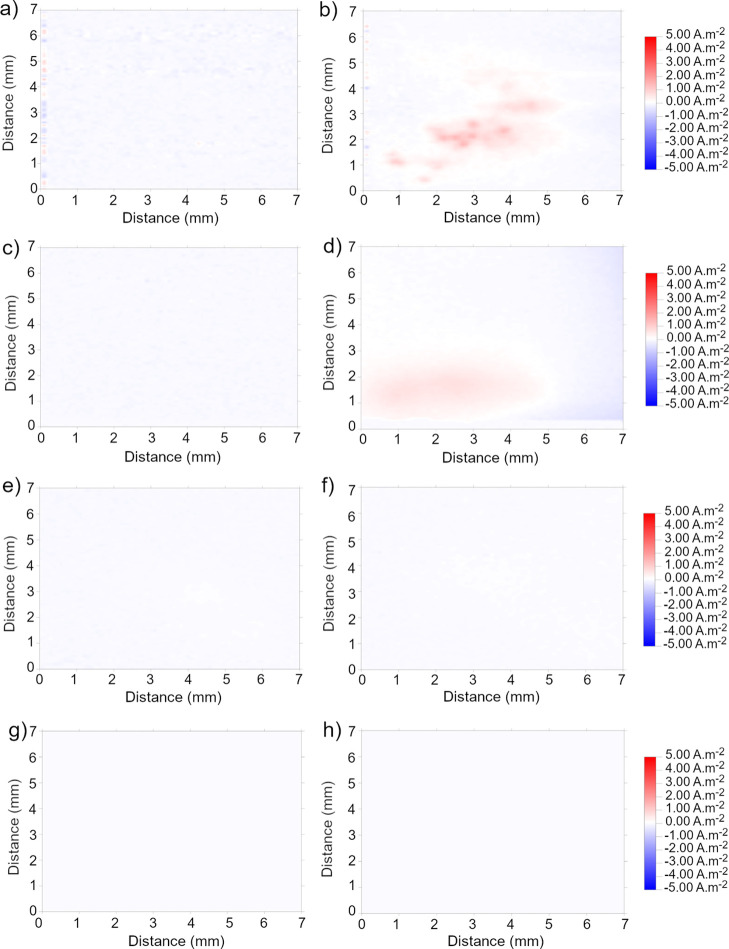
SVET’s map of ionic current over coated HDG steel immersed in 3.5% NaCl for (a,c,e,g) 2 and (b,d,f,h) 72 h (a,b) 0.1 T—0.5 V, (c,d) 0.2 T—0.5 V, (e,f) 0.1 T—3 V, and (g,h) 0.2 T—3 V.

On the other hand, the corresponding maps of the coated surfaces demonstrate a different current density profile (Figure 11). Unlike the bare HDG steel, the electrochemically active sites (either anodic or cathodic) were absent above coatings after 2 h of immersion, indicating that the underlining HDG surface had not been exposed to the electrolyte. However, for both coatings deposited at 0.5 V, after 72 h (Figure 11b,d), the presence of anodic currents could be detected, suggesting that the electrolyte had penetrated through the metal–coating interface. It is worth noting that the current density values for the 0.2 T—0.5 V coatings were slightly less than that of 0.1 T—0.5 V, suggesting that a higher level of TOCN in the deposition provided better barrier characteristics. In contrast to the 0.5 V coatings, the surfaces deposited at 3 V (Figure 11e–h) displayed clear absence of any obvious electrochemical activity over the whole immersion period, indicating that these coatings are inherently more compact and provide higher levels of corrosion resistances.

**Figure 12:**
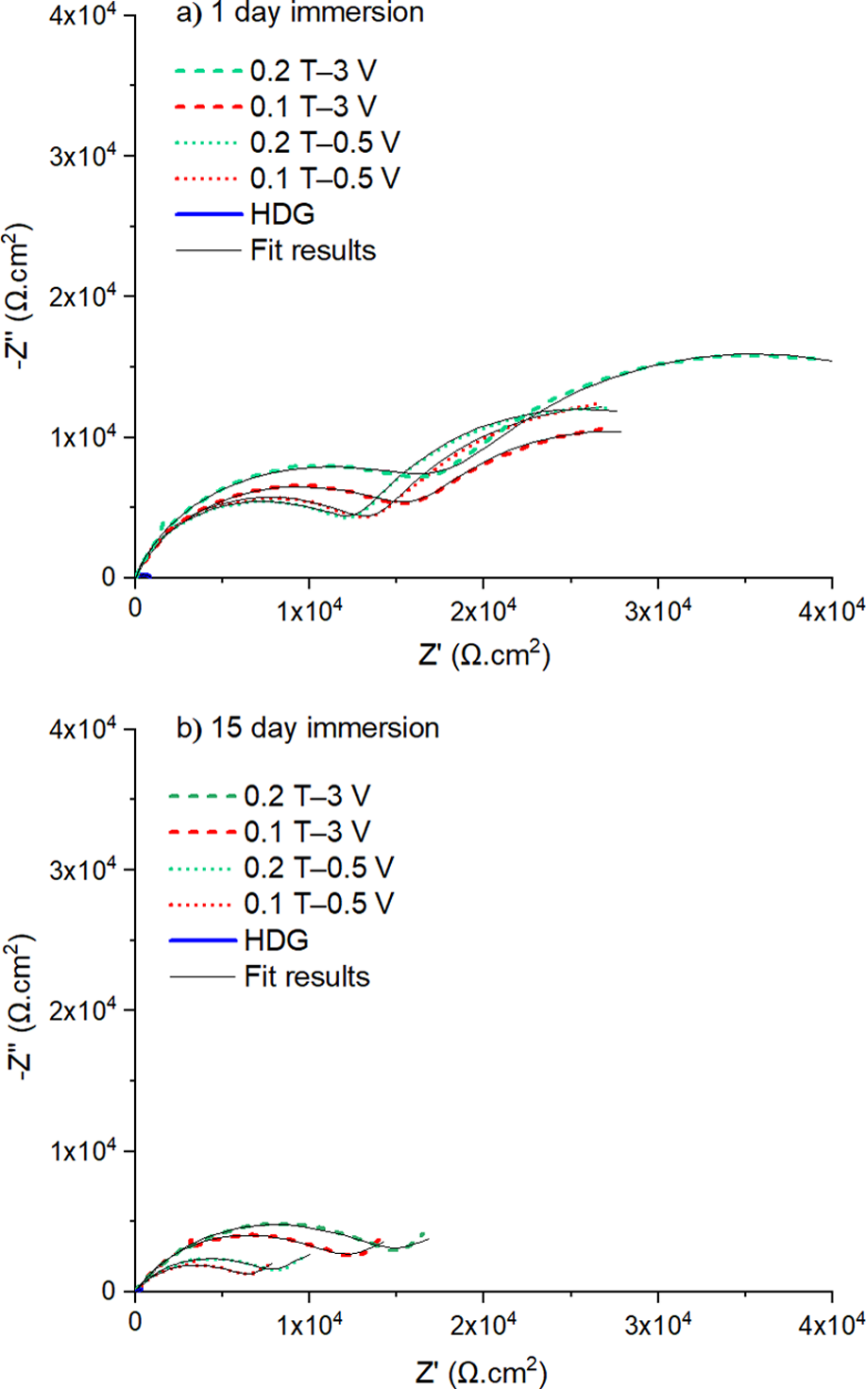
Nyquist plots obtained from the surface of HDG and TOCN–CLP coatings after (a) 1 and (b) 15 days of immersion in 3.5% NaCl.

The electrochemical performances of TOCN–CLPs composite coatings were further investigated by EIS over 15 days of immersion in a 3.5% NaCl solution. Figure 12 presents the Nyquist plots obtained from the uncoated HDG steel and coated surfaces for 1 day (Figure 12a) and coated surfaces for 15 days of immersion (Figure 12b).

As shown in Figure 12a, the Nyquist plots of all four coatings demonstrate a semicircular behavior in the high-frequency region—due to the coating—and this is followed by an incomplete semicircle in the low-frequency domain—related to the electrochemical processes that occur at the coating–metal interface.^64^ The presence of the latter feature indicates that after 1 day of immersion, some electrolyte has already penetrated through the pores/defects in the coatings and has reached the metal–coating interface. With a prolonged immersion for 15 days (Figure 12b), the coating behavior evolves into a single semicircle with an associated low-frequency tail. Such a response indicates that the underlying metal is corroding beneath the surface layer and that the rate is controlled by the diffusion of species through the coating pores/defects.^65^ Moreover, it is worth noting that after 1 day of immersion, the diameter of the semicircle in the high-frequency region—that correlates with coating performance—is largest for the 0.2 T—3 V coating, which suggests that this combination provides the highest level of corrosion protection.^66^ This trend is also visible in the Bode representation of data (Figure S6, Supporting Information), where the highest resistance at the metal–coating interface (*i.e.*, impedance modulus at 10^–2^ Hz) after 1 and 15 days of immersion is obtained for 0.2 T—3 V.

**Figure 13:**
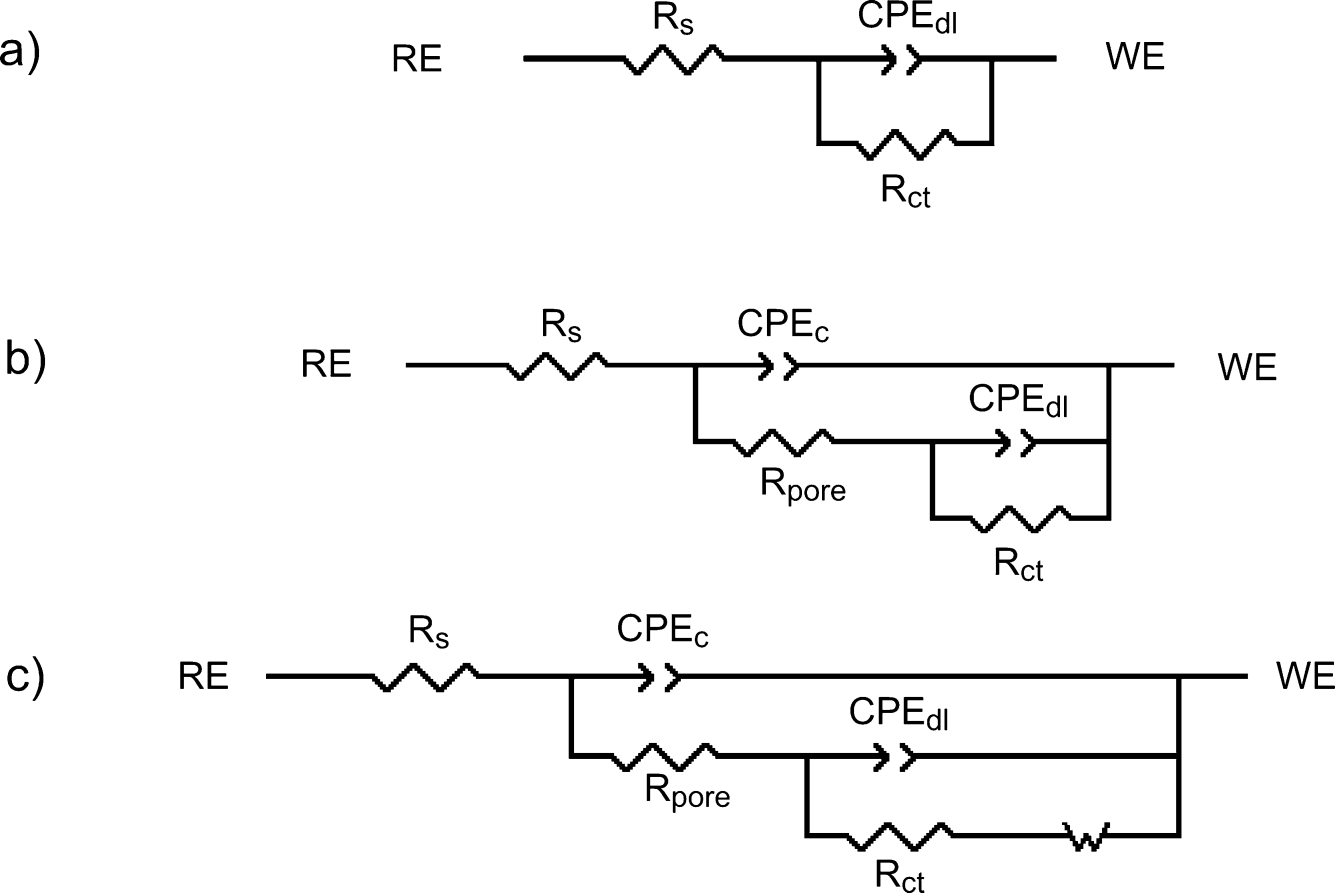
ECs utilized for fitting of the EIS data for (a) HDG steel and (b) coatings after 1 day of immersion and (c) all surfaces after 15 days of immersion in 3.5% NaCl. (RE = reference electrode and WE = working electrode).

Quantitative interpretation of EIS data is often necessary to obtain the values of different components within a complex electrochemical system, and this is obtained by fitting of the experimental data with representative equivalent circuits (ECs). Figure 13 represents the ECs utilized for data fitting of the HDG (Figure 13a) and coated surfaces after 1 day (Figure 13b) and all the surfaces after 15 days of immersion (Figure 13c) in 3.5% NaCl solution. The components within these ECs consist of solution resistance (*R*_s_), nonideal capacitance of coatings (CPE_c_) and coating pore resistance (*R*_pore_), nonideal capacitance at the interface (*i.e.*, double-layer capacitance, CPE_dl_) and charge transfer resistance (*R*_ct_), as well as a Warburg element that considers the diffusion process that occurs at the interface. In the case of HDG steel after 15 days of immersion, the coating elements (Figure 13c) were substituted by elements representing an oxide layer on the HDG surface (CPE_o_ and *R*_o_).

Table 3 lists coating capacitances and the charge transfer resistances obtained from the fitting procedure (a complete list of values is provided in Table S1, Supporting Information). The first noticeable trend in Table 3 is the drop in capacitance values toward the final stages of immersion, which is not a typical trend for immersed organic coatings. In other words, the capacitance of an organic coating usually demonstrates an initial increase upon immersion into the electrolyte—due to the modification of the dielectric constant by electrolyte molecules—until the coatings reaches a saturated state with a relatively constant capacitance.^67^ Considering the hydrophilic nature of cellulose, it is speculated that the drop in capacitance results from dimensional changes that occur within the immersed coatings (*i.e.*, swelling of the cellulose),^68^ and according to the following eq 5(67)
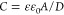
5where *A* is the immersion area of coating, *d* is the coating thickness, and ε and ε_0_ are the dielectric constants of the medium and free space, respectively.

**Table 3:** Selected Parameters for Bare and Coated HDG Samples Obtained from EIS Fitting

time in 3.5% NaCl (days)	sample	coating capacitance (μF·cm^–2^)	*R*_ct_ (kΩ·cm^2^)
1	HDG		0.88
1	0.1 T—0.5 V	14.38	27.02
1	0.2 T—0.5 V	15.93	24.93
1	0.1 T—3 V	18.70	21.17
1	0.2 T—3 V	14.37	34.00
15	HDG		0.22
15	0.1 T—0.5 V	5.35	6.05
15	0.2 T—0.5 V	4.41	7.17
15	0.1 T—3 V	7.82	12.09
15	0.2 T—3 V	7.53	13.68

Another noticeable difference among the samples was the charge transfer resistance (*R*_ct_, Table 3). The comparison of *R*_ct_ values after 1 day of immersion clearly demonstrates that all four coatings provide at least 1 order of magnitude higher *R*_ct_—a sign of hindered electrochemical activity at the metal–coating interface^69^—and between the coatings themselves, the highest resistance was observed with the 0.2 T—3 V coatings (34.00 *cf.* 0.88 kΩ·cm^2^ for HDG steel). However, after 15 days of immersion, the *R*_ct_ for all four coatings show a marked decrease, which indicates that corrosion has further propagated beneath all coated surfaces. Furthermore, the protection capability of 0.5 V samples appears to decrease (the drop in *R*_ct_ throughout immersion) at a faster rate compared to 3 V samples, which implies that the penetration of the electrolyte through the coatings occurs faster with 0.5 than 3 V samples. This correlates with the findings from the SVET, where the appearance of electrochemically active sites starts to slowly become evident after 48 h of immersion for 0.5 V surfaces. Nevertheless, the *R*_ct_ values after 15 days of immersion are still higher in all coatings (by at least an order of magnitude) when compared to the bare HDG steel, indicating the maintenance of some protective functionality for all four coatings throughout the immersion period. Compared to our previous result on pure lignin coatings from a similar softwood KL,^33^ these TOCN–CLP composite coatings offer significantly more reliable protection capabilities that can be readily achieved from aqueous dispersions with minimal volatile organic compound content. Nevertheless, the water swelling characteristics of coatings and its effect on coatings’ mechanical and wet-adhesion characteristic requires further characterization.

## 4 Conclusions

Driven by the need for more sustainable coatings, this study highlights a process for preparation of water-dispersible CLPs and their codeposition with different concentrations of TOCNs. By using an industrial organic solvent (DEGBE), it was shown that colloidally stable particles with submicron sizes can be synthesized through the solvent exchange of organic solutions in water. The CLPs produced display a coalescing characteristic during the drying, which was utilized for formation of compact biopolymeric coatings using EPD. SVET measurements revealed that a higher deposition potential (3 V) results in the formation of more compact coating networks with higher levels of corrosion protection, whereas coatings produced at the lower deposition potential (0.5 V) provide less of a barrier to the electrolyte. EIS measurements (3.5% NaCl, up to 15 days) obtained from HDG and coated surfaces demonstrated the superior protection capabilities of 3 V coatings, as indicated by higher charge transfer resistances (*R*_ct_) for coated surfaces at both the initial (24.93 to 34.00 kΩ·cm^2^ for coatings *cf.* 0.88 kΩ·cm^2^ for HDG steel) and the final stages of immersion (6.05 to 13.70 kΩ·cm^2^ for coatings *cf.* 0.22 kΩ·cm^2^ for HDG steel). Furthermore, the utilized deposition potentials (0.5 and 3 V) and the initial concentration of TOCN in the dispersion (1 and 2 g·L^–1^) appeared to affect the adhesion characteristics and the overall integrity of the coatings.

## Supporting Information Available

SEM micrograph of ambiently dried CLPs; current density profile during EPD (3 V); pH changes during EPD from TOCN suspension; surface cracks observed for 3 V-deposited coatings; coating’s detachment induced by the incision and the tape’s removal; EIS Bode plots obtained from surfaces at different immersion intervals (1 and 15 days, 3.5% NaCl); and quantified values from the fitting of EIS data (PDF)
