# Supplementary material for: Biopolymeric Anticorrosion Coatings from Cellulose Nanofibrils and Colloidal Lignin Particles
Source: ACS Appl Mater Interfaces. 2021 Aug 19;13(34):41034–45. doi: 10.1021/acsami.1c08274 (PMC8414484; doi:10.1021/acsami.1c08274)
Supplement: Supplementary file 1 — am1c08274_si_001.pdf [file am1c08274_si_001.pdf]

Supporting Information

Biopolymeric Anticorrosion Coatings from Cellulose Nanofibrils and Colloidal Lignin Particles

Arman Dastpak[†]*, Philip Ansell*[‡]*, Justin R. Searle*[§]*, Mari Lundström*[†]*, and Benjamin P. Wilson*[†,*]

[†] Hydrometallurgy and Corrosion, Department of Chemical and Metallurgical Engineering (CMET), Aalto University, P.O. Box 16200, FI-00076 Aalto, Espoo, Finland.

[‡] Materials Research Centre, College of Engineering, Swansea University, Bay Campus, Crymlyn Burrow, Swansea SA1 8EN, Wales, United Kingdom.

[§] SPECIFIC, College of Engineering, Swansea University, Bay Campus, Crymlyn Burrow, Swansea SA1 8EN, Wales, United Kingdom.

*E-mail: ben.wilson@aalto.fi

Content: 8 pages, including 6 figures and 1 table.

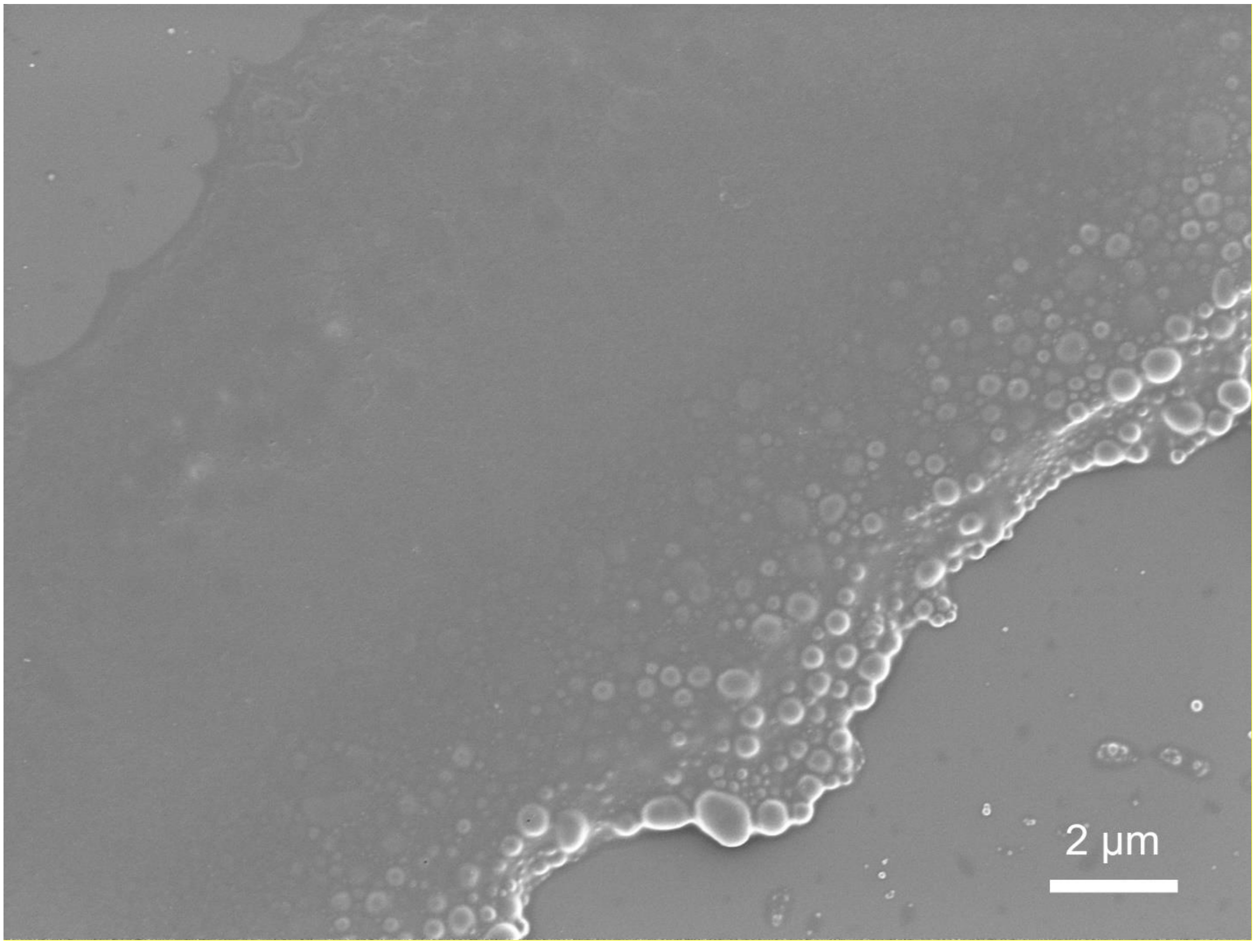

Figure S1. Scanning electron microscopy (SEM) micrographs obtained from the CLPs' surface after drying in ambient temperature for 15 days. The formation of CLPs film suggest that the coalescence is not originated from the heat-treatment process.

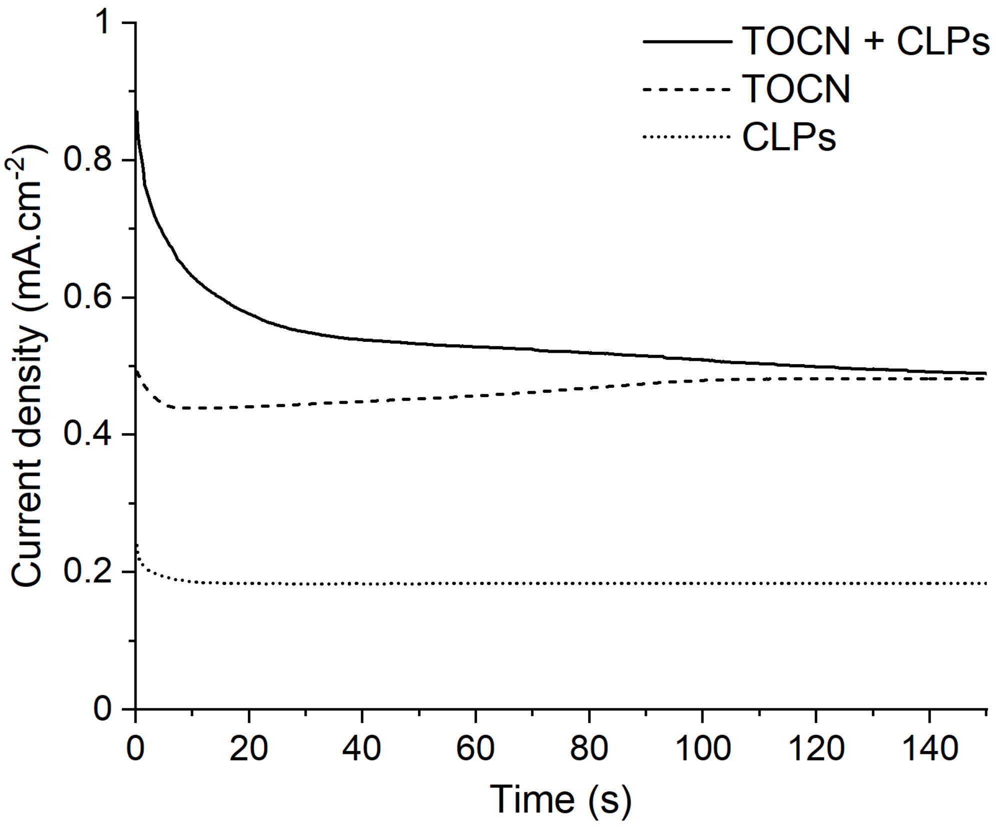

Figure S2. Current density profile during electrophoretic deposition (3 V, 150 s) from TOCN suspension, CLPs dispersion and TOCN-CLPs dispersion. The highest values of current density were obtained during co-deposition of biopolymers.

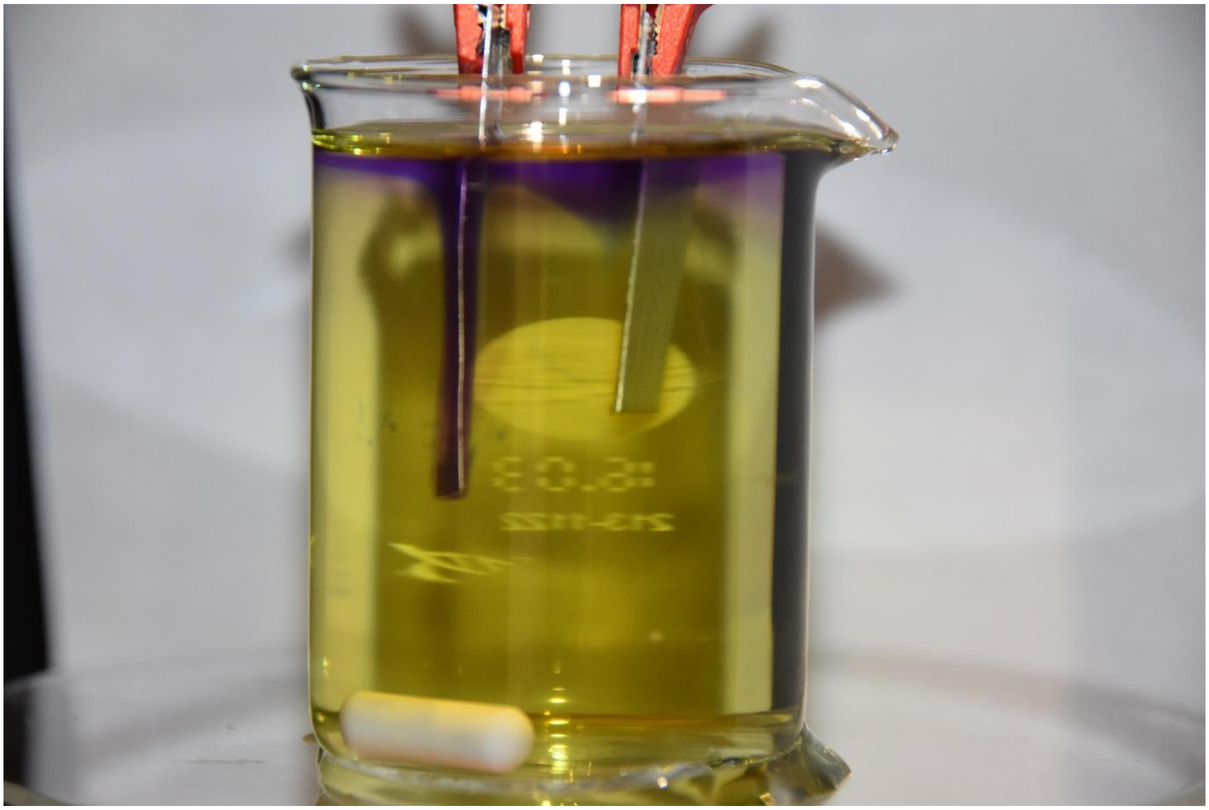

Figure S3. Image obtained at the end of electrophoretic deposition at 3 V from TOCN suspension containing a pH indicator solution. The change of color from green (neutral) to purple (basic) in the vicinity of the cathode (Platinum) suggested an increased local pH due to the hydrogen evolution. Slight change of color to red (acidic) was only observed at the edges of anode (HDG).

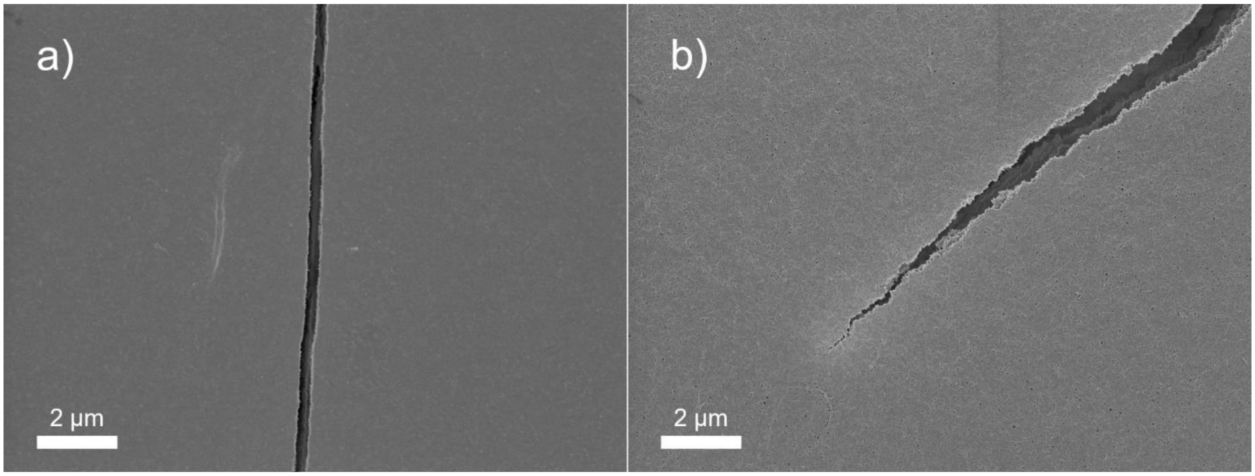

Figure S4. SEM micrographs obtained from the coating surfaces of (a) 0.1 T–3 V and (b) 0.2 T–3 V, demonstrating the presence of local cracks in thicker coatings deposited at 3V.

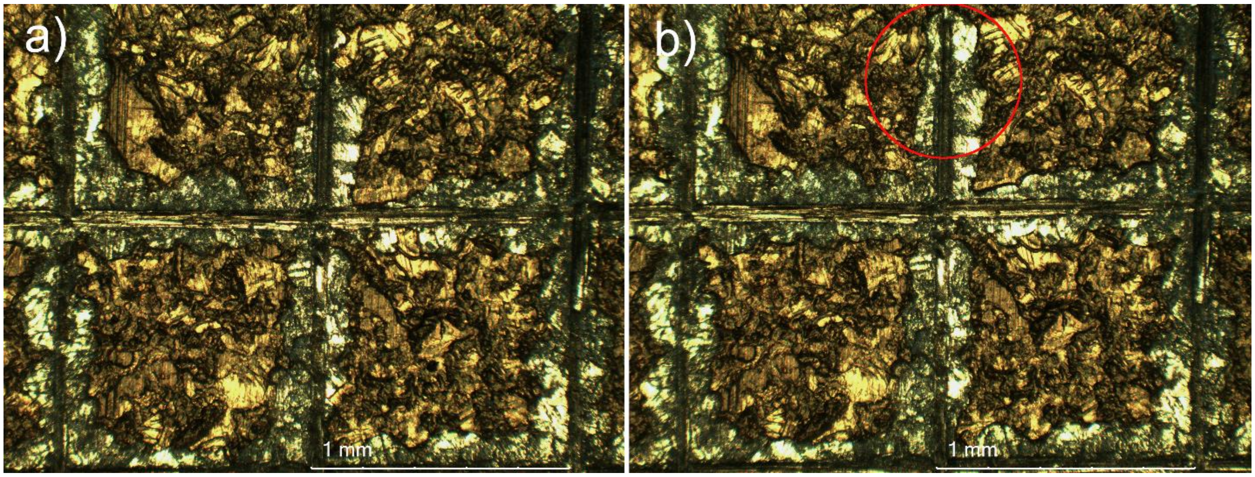

Figure S5. OM images of 0.2 T–3 V's surface after the incision (a) and the tape application and removal (b). The circled area in (b) demonstrates a region where coating was by the adhesive tape. (scale bar: 1 mm)

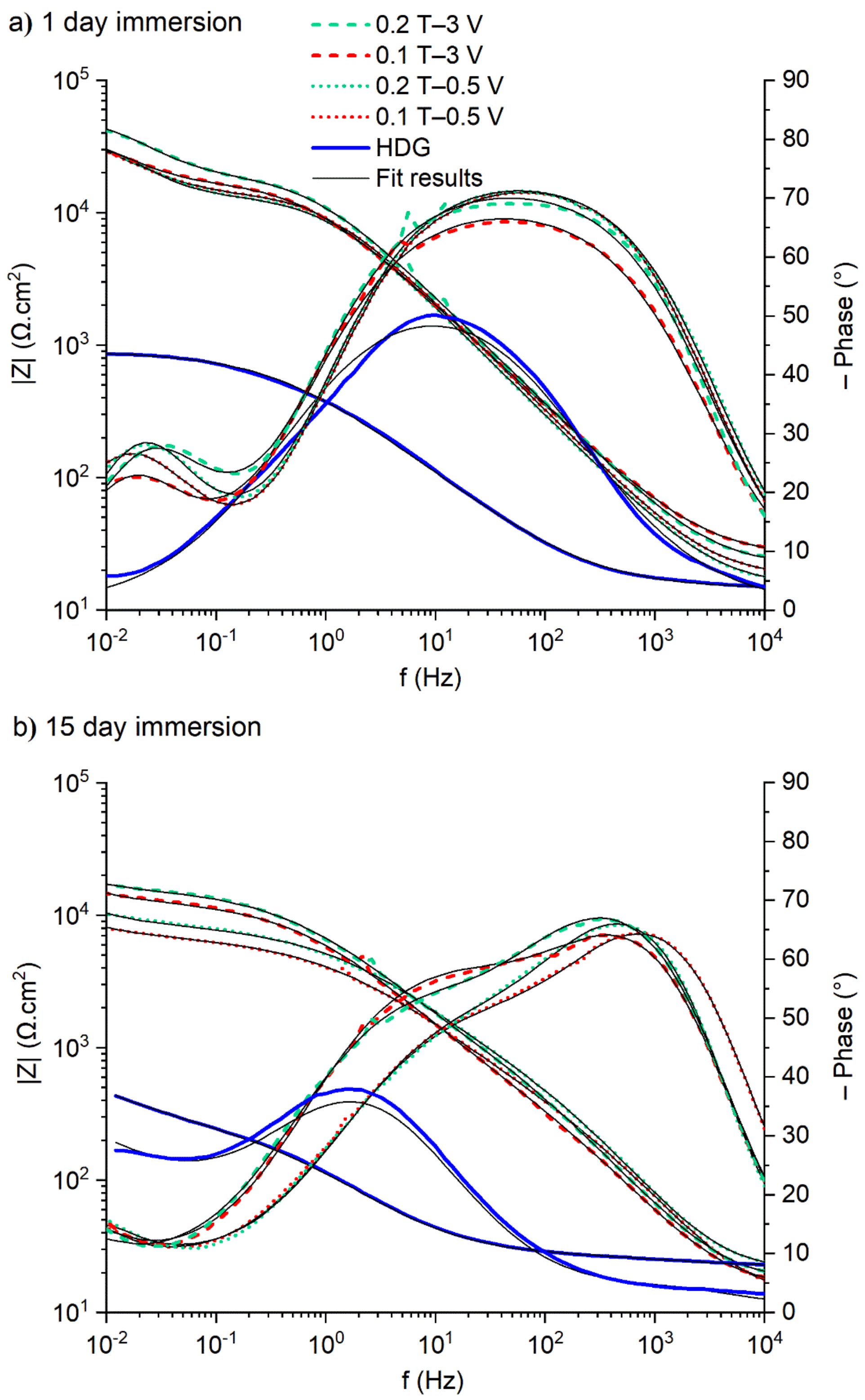

Figure S6. EIS Bode plots obtained from the surface of HDG and biopolymeric coatings after (a) 1-day and (b) 15 days immersion to 3.5% NaCl.

Table S1. Quantified values obtained from the fitting of EIS data using the ECs.

Days in 3.5% NaCl	Sample	R_s (Ω)	R_{pore} ($k\Omega.cm^2$)	CPE_c Y ($\mu F.cm^{-2}$)	CPE_c n	R_{ct} ($k\Omega.cm^2$)	CPE_{dl} Y ($mF.cm^{-2}$)	CPE_{dl} n
1	HDG	14.4	-	-	-	0.88	0.58	0,66
1	0.1 T–0.5 V	17.7	14.82	14.38	0.83	27.02	0.46	0.90
1	0.2 T–0.5 V	15.2	14.07	15.93	0.83	24.93	0.43	0.93
1	0.1 T–3 V	25.4	18.08	18.70	0.78	21.17	0.55	0.92
1	0.2 T–3 V	21.4	20.51	14.37	0.81	34.00	0.25	0.90
15	HDG	22.4	0.006[a]	413[b]	0.70[b]	0.22	1.89	0.70
15	0.1 T–0.5 V	13.3	0.48	5.35	0.89	6.05	0.04	0.62
15	0.2 T–0.5 V	21.1	0.71	4.41	0.91	7.17	0.03	0.63
15	0.1 T–3 V	15.7	0.55	7.82	0.88	12.09	0.03	0.65
15	0.2 T–3 V	17.3	1.25	7.53	0.87	13.68	0.02	0.66

[a] Resistance of the oxide layer (R_o) on HDG

[b] Capacitance elements for the oxide layer (CPE_o)
